# Dr. G. V. Black’s Conclusions Reviewed

**Published:** 1897-12

**Authors:** George A. Maxfield

**Affiliations:** Holyoke, Mass.


### DR. G. V. BLACK'S CONCLUSIONS REVIEWED.[1]

BY GEORGE A. MAXFIELD, D.D.S., HOLYOKE, MASS.

In attempting to discuss the subject assigned for this evening, I want first to express my appreciation of Dr. Black's work as presented to us in the article in question. A brief examination of the tables given must convince one of the immense amount of labor involved and the scientific accuracy with which the work was carried on. The facts presented appeared to shatter the whole foundation on which we had built. What we have considered and taught as scientific facts, according to Dr. Black, are simply traditions and erroneous fallacies. We cannot question these facts. They are scientific truths.

We can no longer seek to find coarse and unpalatable foods with the expectation of increasing the amount of the lime salts in the teeth. The vast amount of literature on this subject, to which many of us have added, advocating that of which we knew nothing, because we simply accepted the "traditions of the Fathers," must now be cast into the flames. Would that the fallacies they have created could as easily be destroyed and our minds be left free to discuss Dr. Black's conclusions without bias and with a full understanding of the facts he has presented. Personally, I fully agree with many of Dr. Black's conclusions, while the ones proposed for this discussion are at direct variance with my clinical experience.

On page 354, almost at the beginning of his paper, he says, "Two well-known facts have seemed to argue against the interpretation that the clinical appearances mean soft or softening teeth. First, the teeth are the hardest structures of the body, and are of the lowest vital endowment as to self-repair, and are therefore the

---

[1] Read before The New York Institute of Stomatology, October 5, 1897.

slowest in vital changes. Second, caries, according to present accepted theories, attacks the teeth from without, acts upon the teeth, beginning upon the outside, and *presumably the physical or vital condition of the teeth themselves* has little to do in the case. Both of these propositions, if admitted, seem to argue that this melting down of the teeth is dependent on some factor or factors outside of and practically independent of the physical or vital conditions of the tissues of the tooth." (Italics mine.)

After a careful study of Dr. Black's paper, with the light of my clinical experience, I cannot admit this proposition. It is on this presumption that Dr. Black based his conclusion, that "caries of the teeth is not dependent upon any condition of the tissues of the teeth, but on conditions of their environment."

With the knowledge of Dr. Black's long clinical experience and the results obtained by his studies of the "Physical Characteristics of the Human Teeth," it is surprising that he should announce this as a logical conclusion. To my mind the study of this paper gives convincing proof that the physical or vital conditions of the tissues of the teeth are an important factor of caries, and to support this claim I present several quotations.

On page 416 we find the following: "The logical inference is that the cause of the differences in the liability of individuals to caries of the teeth is something in the constitution operating through the oral fluids, and acting upon the active cause of caries, hindering or intensifying its effect." The first question to consider is, do the oral fluids contain a principle or substance which by its presence renders the teeth immune from caries or exerts deterrent action upon the caries-producing organisms? On page 418 he advances the following in support of this theory: "This hypothesis is further supported by the well-confirmed observation that the opening of a carious cavity by accident or intentionally by breakage of overhanging walls has a marked deterrent effect upon the rate of progress of the carious process." Immediately following he answers this by saying, "This again may in some degree be explained by the simple absorption of these products by the flow of saliva." To obviate this latter objection to his theory he says, "But this kind of dissipation of the products would be measurably the same for different individuals, while the observation is that the effect of the dissipation is greatly different in different individuals." If instead of this latter he had said, This kind of dissipation of the products would be measurably the same in the different cavities in the same mouth,—which is a fact well known to all careful observers,—it would have been as applicable to one side of the argument as the other. Again, on page 417, "It seems to be admitted by all who have examined the subject from the stand-point of the bacteriologist that the caries-producing organisms are present in every mouth, whether caries occurs or not. I have myself made cultures from the saliva of many people, those in whose teeth caries was making sad havoc and those in whose teeth no caries whatever appeared, and must say that I have found no mouth that was free from the caries-producing organisms." If the oral fluids contain this "deterrent principle or substance," how is it these caries-producing organisms flourish in every human mouth, regardless of any carious process in the teeth?

Accepting the theory which has been so ably demonstrated by Drs. Williams, Black, Andrews, Miller, and many others, that caries of the teeth is the result of the action of micro-organisms, let us consider whether there can be any change in the teeth that may render them at times less liable to the ravages of these organisms. Are there any differences in the structure of the teeth of different individuals, or in the various teeth of the same individual? Do physiological changes occur in the teeth? Let us read Dr. Black's views.

On page 391, referring to the specific gravity: "But there is a difference between the individual teeth of the same person that is much greater than the difference in averages of the teeth of different persons. Take the first number in the general table, No. 142,—sixteen teeth from one person. The upper right central incisor has a specific gravity of 2.085, while the left, standing beside it, and apparently under exactly similar conditions of development and nutrition, has a specific gravity of 2.103, a difference of eighteen-thousandths of a volume." The wide variation in this individual amounted to sixty-five-thousandths of a volume. Reference to the table show the per cent. of lime salts of the upper right second molar to be 65.12, and the adjoining third molar to be 62.73, a difference of 2.39 per cent.

On page 393: "From the study of these exhibits it is found that there is practically a continuous increase in the density of the teeth, and in the percentage of lime salts they contain, from youth to old age. . . . Beginning with 62.26 per cent. for the average age of eleven years, it increases to an average of 64.56 per cent. for the average age of sixty-three years. This is an increase of 2.3 per cent. This result confirms the hypothesis arrived at from clinical observation, namely, that the density of the teeth increases from childhood to adult age. But it does more. It shows the increase continues practically during life, and that in this respect the teeth are similar to the bones." Again, on page 414, referring to pulpless teeth, "The parting of enamel from the dentine seems to be peculiar to pulpless teeth and to those in which the pulp has been long calcified so as to cut off the *nutrition of the dentine*."

The above shows that Dr. Black believes that the vital action is not confined to the pulp, but extends throughout the whole tooth.

On page 392 he says, "After the work I have done with the teeth I have come to regard the organic matrix of the tooth as much more important than I had formerly considered it. The condition of the organic matter seems to have much to do with the strength of the teeth, and is a matter that needs further investigation."

Clinical observation demonstrates to us that there are changes occurring in the teeth, a continual retrogression, at times rapid, while at other times there is a decided reaction, and the tooth recovers its normal tone. From Dr. Black's demonstrations we learn that these changes must be in the organic structure of the teeth and not in the amount of the lime salts. There are differences in teeth, and we cannot express these differences in better terms than we have always used, "hard teeth," "dense teeth," "frail teeth," "soft teeth," "chalky teeth." As this difference must be wholly in the organic matrix of the tooth, therefore the logical inference is they are the result of vital influences. The liability of caries is much greater in those teeth classed as soft than those classed as hard. Clinical observation also demonstrates that pulpless teeth are less liable to caries than teeth in the same mouth containing living pulps, and when caries begins the progress is much slower.

As to the environment, I quote from page 418, where he refers to the artificial production of caries outside of the mouth: "This observation I have myself verified, and the difference in the rapidity of the progress thus produced artificially in the test-tubes and that occurring under what may be called natural conditions within the human mouth contrasted. I have never seen caries in the mouth progress with such rapidity under any conditions whatever, either in teeth with living or dead pulps. Perhaps some difference may be explained by the absorption and removal of the acid formed by the micro-organisms through the continuous flow of the oral fluids, thus preventing the same degree of concentration of the acid products. But at best this can be but a very partial explanation of the phenomena observed, and is no explanation whatever of the differences in the susceptibility of the teeth of different individuals. It is known that micro-organisms do not grow so well in the concentration of the lactic acid which occurs in our culture-tubes, and that if this is allowed too great a concentration their growth ceases entirely. Yet they will penetrate dentine farther in the same length of time in the tubes than they will in the mouth.

"If this proposition is correct, we must conclude that there is some deterrent force operating within the human mouth that is not present in the test-tubes, a force of some kind, or of some nature, that acts to hinder the destructive effects of the products of the micro-organisms, either by restraining the growth of the organisms by hindering the production of their typical acid excretions, or by limiting the effects of the acid excreted by the organisms. Such an effect seems not to be produced by any condition of the teeth themselves connected with their vitality, since the observation applies with *equal force to pulpless or dead dentine* as to *living dentine.* Then this power, or deterrent force, is extraneous to the teeth; its action is from without."

This is the strongest argument advanced by Dr. Black in support of his conclusion. In the culture-tubes there can be no difference between pulpless teeth and those in which there were living pulps. *In either case, can it be other than dead dentine?*

When we consider this, together with the question already raised as to the possibility of the oral fluids containing the deterrent principle or substance, and the caries-producing organisms at the same time, I hardly think the argument is sustained.

From these studies we arrive at the following logical conclusions, namely, that the physiological and pathological changes which occur in the organic structure of the teeth are an important factor in the progress of caries.

I would not belittle the work of Dr. Black one iota, for I sincerely believe that as he continues his investigations he will discover facts verifying our clinical observations, and other facts which will enable us by natural physiological processes to produce changes in the organic structure of the teeth, whereby they will be able to repel the caries-producing organisms.

